# Effect of a New Synergistic Combination of Low Doses of Acetylsalicylic Acid, Caffeine, Acetaminophen, and Chlorpheniramine in Acute Low Back Pain

**DOI:** 10.3389/fphar.2019.00607

**Published:** 2019-06-20

**Authors:** Victor A. Voicu, Constantin Mircioiu, Cristina Plesa, Mariana Jinga, Vasile Balaban, Roxana Sandulovici, Ana Maria Costache, Valentina Anuta, Ion Mircioiu

**Affiliations:** ^1^Department of Clinical Pharmacology, Toxicology and Psychopharmacology, Faculty of Medicine, “Carol Davila” University of Medicine and Pharmacy, Bucharest, Romania; ^2^Doctoral School, “Carol Davila” University of Medicine and Pharmacy, Bucharest, Romania; ^3^Department of Neurology, “Dr. Carol Davila” Central Military Emergency University Hospital, Bucharest, Romania; ^4^Department of Internal Medicine and Gastroenterology, Faculty of Medicine, “Carol Davila” University of Medicine and Pharmacy, Bucharest, Romania; ^5^Internal Medicine and Gastroenterology Clinic, “Dr. Carol Davila” Central Military Emergency University Hospital, Bucharest, Romania; ^6^Department of Applied Mathematics and Biostatistics, Titu Maiorescu University, Bucharest, Romania; ^7^Department of Clinical Research, CEBIS International, Bucharest, Romania; ^8^Department of Physical and Colloidal Chemistry, Faculty of Pharmacy, “Carol Davila” University of Medicine and Pharmacy, Bucharest, Romania; ^9^Department of Biopharmacy and Pharmacokinetics, Titu Maiorescu University, Bucharest, Romania

**Keywords:** low back pain, synergistic combination, lowest-dose pain relief, Algopirin®, safer drug use

## Abstract

The present paper continues a more complex research related to the increased synergism in terms of both anti-inflammatory and analgesic effect obtained by the addition of chlorpheniramine (CLF) to the common acetylsalicylic acid (ASA), acetaminophen (PAR), and caffeine (CAF) combination. This synergistic effect was previously highlighted both *in vitro* in rat models and *in vivo* in the treatment of migraine. The aim of the research was to further evaluate the analgesic effect of a synergistic low-dose ASA–PAR–CAF–CLF combination in the treatment of low back pain, in a parallel, multiple-dose, double-blind, active controlled clinical trial. A number of 89 patients with low back pain of at least moderate intensity were randomly assigned to receive Algopirin^®^ (ALG), a combinational product containing 125 mg ASA, 75 mg PAR, 15 mg CAF, and 2 mg CLF, or PAR 500 mg, a drug recognized by American Pain Society as “safe and effective” in the treatment of low back pain. One tablet of the assigned product was administered three times a day for seven consecutive days. The patients evaluated their pain level using a Visual Analog Scale prior to administration, and at 1, 2, 4, and 6 h after the morning dose. Time course of effect was similar in structure and size for both treatments. Pain relief appeared rapidly and steadily increased over 4 h after drug administration. Differential pain curves of ALG and PAR were very similar and comparable with the previously determined ALG analgesia pattern in migraine. Differences between the daily mean pain scores were not statistically significant for the two treatments. Similar results were obtained for the Sum of Pain Intensity Differences (SPID) for 0–4 h and 0–6 h intervals as well as for the time course of the proportion of patients with at least 30% and at least 50% pain relief. In conclusion, in spite of very small doses of active components, ALG proved equally effective to the standard low back pain treatment and therefore a viable therapeutic alternative, mainly for patients with gastrointestinal and hepatic sensitivity.

**Trial Registration:** www.ClinicalTrials.gov, identifier EudraCT No.: 2015–002314–74.

## Introduction

Low back pain (LBP) affects a large number of people in developed countries and, following the associated disability, has important consequences on the health system and economy (Frymoyer, 1988; Liddle et al., 2007). In fact, LBP is the fifth most common reason for all visits to physicians for clinical diagnosis, treatment, and evaluation in the United States (Hart et al., 1995; Deyo et al., 2006).

LBP is usually divided into acute LBP (i.e., persisting for less than 6 weeks), sub-acute LBP (i.e., persisting between 6 and 12 weeks), and chronic LBP (i.e., persisting for more than 12 weeks) (van Tulder et al., 2006). In some cases, LBP is self-limited without medical treatment (Carey et al., 1996), but most frequently, pain and disability are persistent for a long time (Pengel et al., 2003) and require more or less intensive treatments.

### Low Back Pain Therapy

The common therapy to reduce pain, inflammation, and functional disability includes nonsteroidal anti-inflammatory drugs (NSAIDs), PAR, corticosteroids, and various opioids.

#### Nonsteroidal Anti-Inflammatory Drugs

NSAIDs are the most widely used drugs in the world and their use in the treatment of LBP makes no exception (Matsumo et al., 1991). In fact, a Joint Guideline of the American College of Physicians and the American Pain Society makes the recommendation that “for most patients, first-line medication options are acetaminophen or nonsteroidal anti-inflammatory drugs (NSAIDs)” (Chou et al., 2007).

A systematic review of the clinical trials with NSAIDs in LBP (Koes et al., 1997) analyzed 26 randomized clinical trials. Pooled odds ratios in 10 studies comparing NSAIDs with placebo in similar patient groups and using similar outcome measures for 1 week was found to be 0.53 (95% CI, 0.32–0.89), i.e., a statistically significant effect. It is however important to note that the measured endpoints were sufficiently different between studies that the risk of doubtful conclusions cannot be ignored.

In a later report from 2000 (van Tulder et al., 2000), the analysis included a total of 51 trials (6,057 patients). The pooled relative risk for global improvement after 1 week was 1.24 (95% CI, 1.10–1.41), indicating a statistically significant effect in favor of NSAIDs compared to placebo. The results indicated that there is moderate evidence that NSAIDs are not more effective than other drugs for acute LBP and there is strong evidence that various types of NSAIDs are equally effective for acute LBP. Later, the same Cochrane group, after extending the analysis to 65 studies, draw the same conclusions, adding that, however, effect sizes are small (Roelofs et al., 2008).

More recently, a meta-analysis (Enthoven et al., 2016) revealed that the above conclusions remain valid also in the case of chronic LBP. When they included only studies with low bias risk, the differences in effect between NSAIDs and placebo were further reduced (7 points on a 100-point scale for pain intensity in trials lasting from 9 days to 16 weeks) and no differences in efficacy between different NSAID types were identified (Enthoven et al., 2017).

#### Nonsteroidal Anti-Inflammatory Drugs *vs.* Acetaminophen

Four studies compared one or more types of NSAIDs with PAR, concluding that there is moderate evidence that NSAIDs are equally effective with PAR for acute LBP (Evans et al., 1980; Wiesel et al., 1980; Milgrom et al., 1993; Nadler et al., 2002). However, NSAIDs were associated with more side effects compared to PAR (relative risk, 1.76; 95% CI, 1.12–2.76; *N* = 309).

In LBP, PAR is considered safer than NSAIDs, since NSAIDs are often associated with upper gastrointestinal tract bleeding/perforation (Hernández-Díaz and Rodríguez, 2000). However, PAR, for its part, is associated with agranulocytosis (Gursoy et al., 1996)asymptomatic elevations of aminotransferase levels at dosages of 4 g/day even in healthy adults (Rahme et al., 2002), although the clinical significance of these findings is uncertain (Watkins et al., 2006).

#### Acetylsalicylic Acid

ASA was compared to other drugs in LBP in an old study on 45 patients admitted to a military hospital (Wiesel et al., 1980). 625 mg ASA capsules were administered four times a day for 2 weeks and compared with 100 mg phenylbutazone capsules administered under the same dosage regimen. Differences between the effects of the two treatments were not significant. Another study compared three 300-mg capsules of ASA to two 500-mg capsules of PAR in the same year (Evans et al., 1980). Both treatments were administered four times a day for 1 week. There was moderate evidence that ASA is equally effective for pain relief and global improvement compared with PAR for acute LBP.

Combinations of ASA and other compounds in the LBP treatment were tested since the late 1980s. Hence, the following treatments were administered three times a day to groups of 40 patients each: i) mefenamic acid 500 mg; ii) chlormezanone 100 mg and paracetamol 450 mg; and iii) ethoheptazine 75 mg, meprobamate 150 mg, and ASA 250 mg. The number of patients reporting no pain after 1 and 7 days were i) 21, ii) 23, and iii) 20. The number of patients reporting adverse effects in the study was i) 9, ii) 10, and iii) 16 (Sweetman et al., 1987).

Aside from the fact that treatments had approximately the same effect, it is important to note that “no pain” can rarely be achieved, only after several weeks of treatment, and only for patients with moderate initial pain. Therefore, pain relief to less than 70% or to less than 50% of the initial value could therefore be a more appropriate marker of effect.

In all the above studies implying treatments longer than a few days, side effects that cannot be neglected also appeared (Lanas et al., 1997).

### Synergy-Based Therapeutic Approaches

Synergistic drug combinations have been envisaged to be a promising approach to treat in pain treatment, since the complementary mechanism of action of the components lead to an effect that is superior to the additive effects of the individual constituents. In fact, the term “synergy” itself, originating from the Greek word meaning “working together,” perfectly resumes how “cooperation” between different active moieties leads to a combined boost in drug efficacy.

ASA–PAR–CAF is a well-known and may be the most representative example of synergism (Bosse and Kühner, 1987). A meta-analysis of the effect of ASA–PAR–CAF combination involving more than 10,000 patients has been carried out more than 30 years ago (Laska et al., 1984). The overall pooled relative potency estimates of 26 clinical trials highlighted that a 40% lesser analgesic dose is required for obtaining the same response when administered in combination with CAF. The combination was subsequently reported to be superior in the treatment of headache both to placebo and to sumatriptan (Goldstein et al., 2005), ibuprofen (400 mg) (Goldstein et al. 2006), ASA–PAR combination, as well as to ASA, PAR, and CAF control therapies (Diener et al., 2005). The optimal dose ratio of ASA–PAR–CAF was found to be 1:1:0.25, with the minimum dose of CAF being 50 mg (Diener et al., 2005; Temple et al., 2017).

Combinations containing less than 250 mg PAR, 250 mg ASA, and 50 mg CAF per dosage form were extensively studied as analgesics in migraine in the last 40 years (Wenzel et al., 2003; Reddy, 2013). In 1993, FDA recommended these combinations as “recognized as safe and effective” analgesics (Hersh et al., 2000) and the American Academy of Neurology considered them as first-line migraine treatment (Silberstein, 2000). In fact, the ASA–PAR–CAF combination is widely used for the treatment of pain, under the name Excedrin^®^ in the US and Anadin Extra^®^ in UK.

Addition of antihistamines to the ASA–PAR–CAF combination led to a new class of anti-inflammatory and analgesic drugs with even stronger synergism (Malec, 1987). Hence, chlorpheniramine (CLF), although not presenting intrinsic analgesic effect (Rumore and Schlichting, 1986; Raffa, 2001), significantly potentiates both the anti-inflammatory and analgesic effect of the ASA–PAR–CAF combination. This strong potentiation effect was underlined in our previous studies both *in vitro*, in analgesia and carrageenan-induced rat paw inflammation models, as well as *in vivo* in the treatment of mild algic syndrome (Voicu et al., 2016).

The subsequent researches focused on evaluation of the ASA–PAR–CAF–CLF low-dose combination in the treatment of migraine. Thus, clinical studies (Blendea et al., 2011; Enache et al., 2012) proved the noninferiority of a unique dose of Algopirin^®^ (ALG), an authorized combination of the four active containing 125 mg ASA, 75 mg PAR, 15 mg CAF, and 2 mg CLF (Voicu et al., 2016; Voicu et al., 2017a) vs. Excedrin^®^, a fixed combination drug containing 250 mg ASA, 250 mg PAR, and 65 mg CAF. The extension study (Voicu et al., 2017b) also proved the superiority of two tablets of ALG vs. one tablet of Excedrin^®^, though the doses of active components in the ALG treatment remained lower than in the case of Excedrin^®^.

The present study aims to extend our research regarding the efficacy of the synergistic low-dose ASA–PAR–CAF–CLF combination in the treatment of acute LBP, in a parallel, multiple-dose, double-blind, active controlled clinical trial, for the purpose of offering a therapeutic alternative with comparable efficacy to PAR 500 mg.

## Materials and Methods

### Patients

The clinical trial was conducted in the “Dr. Carol Davila” Central Military Emergency University Hospital, Bucharest, Romania. The study conformed with the Helsinki Declaration of 1964, as revised in 2013, with the International Conference on Harmonization (ICH) Good Clinical Practice regulations, as well as the Joint Clinical Practice Guideline from the American College of Physicians and the American Pain Society on the diagnosis and treatment of LBP (Chou et al., 2007).

The study protocol (EudraCT number: 2015-002314-74) was approved by the National Agency for Medicines and Medical Devices (approval number 30523E/04.04.2016) and the National Bioethics Committee for Medicines and Medical Devices (approval number 124/2016). All participants gave written informed consent prior to study participation and were instructed by specialized personnel on how to record the characteristics of their back pain. All patients were allowed to terminate their participation in the trial at any time, without restrictions.

A number of 89 male and female patients (37 males and 52 females), aged between 18 and 65 years, were enrolled in the study by neurology or internal medicine specialists at the clinical facility. The enrolled subjects fulfilled the inclusion criteria specified in the protocol, having at least moderate pain intensity (with a score higher than 40 on the 1–100 units Visual Analog Pain Intensity Scale). Patients were excluded if they were under ongoing treatment with ASA-, PAR-, CAF-, or CLF-containing drugs as well any other prescription or nonprescription analgesics, antirheumatic, or anti-inflammatory drugs in the last 4 days or if they were under ongoing treatment with anticoagulant agents. Exclusion criteria also included special physiological conditions (such as pregnancy, breastfeeding); hypersensitivity to ASA, PAR, or CAF; alcohol or drug abuse; different diseases [gastrointestinal ulcer, bleeding diathesis, glucose 6-phosphate dehydrogenase deficiency, asthma, liver disease (aspartate aminotransferase, alanine aminotransferase, and total bilirubin more than two times the upper limit of normal), Gilbert syndrome or hyperthyroidism, preexistent renal impairment (estimated creatinine clearance less than 40 ml/min as calculated by the Cockcroft–Gault equation)]; or any major neurological disorders.

During the initial screening and in treatment days 1, 3, and 4, patients responded to the Roland–Morris Questionnaire (RMQ), a self-rated physical disability measure on a 24-point scale.

### Study Medication

All study medication, e.g., the PAR 500 mg tablets used as active comparator as well as the investigated drug Algopirin^®^ (ALG) (a combinational product formulated as tablets containing 125 mg ASA, 75 mg, PAR, 15 mg CAF, and 2 mg CLF) were provided by Polisano Pharmaceuticals S.R.L., Sibiu, Romania.

The double-blind character of the study was ensured by utilizing matched trial supplies, identical in both taste and appearance (color, shape, and size).

### Study Design

The study was designed as a noninterventional, randomized, parallel, multiple-dose, double-blind, noninferiority clinical trial, comparing the effectiveness of ALG tablets (125 mg ASA, 75 mg PAR, 15 mg CAF, and 2 mg CLF) versus PAR 500 mg tablets, a drug recognized by the American Pain Society as “safe and effective” in the treatment of LBP.

Each participant in the study received a tablet of the assigned product three times a day, for seven consecutive days. The patients evaluated their pain level using a Visual Analog Scale prior to administration, and at 1, 2, 4, and 6 h after the morning dose in the first five as well as in the last day of the study.

No other drug intake was allowed in the first 4 h after study medication administration. Patients were allowed to use rescue medication (PAR 500 mg) only 4 h after the administration of the study medication and were not allowed to drink coffee or CAF-containing beverages within 2 h before and after administration of the trial medication.

### Efficacy Measurement

Pain intensity was assessed on a horizontal 100-mm Visual Analog Pain Intensity [VAS(PI)] scale labeled No Pain (0 mm) on the left end and Worst Pain Imaginable (100 mm) on the right end. In the screening visit, the investigator provided to each of the enrolled patients a standardized explanation on how to evaluate and record the VAS(PI) score, using a written explanatory text.

Patients were required to record in a diary the date and time of drug administration and pain intensity on the VAS(PI) scale baseline immediately before each trial drug administration, as well as at 1, 2, 4, and 6 h after the morning dose on days 1, 2, 3, 4, 5, and 7 of the clinical trial. The investigator reviewed the completed diary with the patient in order to ensure that all the significant information, including safety and tolerability issues, had been documented.

### Endpoints

The primary endpoint of the trial was considered the mean pain intensity score evaluated on the VAS(PI) scale after five treatment days.

Secondary endpoints were:

- Proportion of patients who achieved at least 50% pain relief at different intervals after administration [evaluated on VAS(PI) by linear interpolation between consecutive observation time points];
- Time-weighted Sum of Pain Intensity Differences (SPID) relative to baseline for 4 or 6 h after intake of the medication, evaluated on a daily basis;
- Hazard ratio of the percentage of patients with at least 50% pain relief as a function treatment duration;
- Safety assessment.

### Statistical Analysis

The statistical analyses as well as graphical representation of data were performed using GraphPad Prism 7 (GraphPad Software Inc., La Jolla, CA, USA) software. Statistical comparisons of different numerical data sets (such as daily mean pain scores as well as the SPID_0-4h_ and SPID_0-6h_ values) were performed using Student’s *t* test. Differences were considered statistically significant when *p* values were <0.05.

For the categorical variables, the comparisons were performed using chi-square test (significance level, *p* < 0.05).

The percentage of patients in the two treatment groups with pain reduced to less than 70% and 50% from baseline as a function of the number of days of treatment was compared using log-rank test.

### Sample Size Estimation

In clinical trials concerning analgesia, the sample size is usually estimated in order to ensure 0.8 power to detect differences between treatments, considering the significant difference in clinical success rate 20% based on VAS(PI) evaluation (Diener et al., 1999).

Considering the probability of type I error *α* = 0.10, for type II error *β* = 0.20, a coefficient of variation $CV=\frac{\sigma }{\mu }*100$ of 60%, and a normal distribution of SPID values, a necessary number *n* = 81 subjects was obtained. Estimating a 10% proportion of outliers or premature withdrawals, the number of patients to be enrolled was set at 90.

## Results

### Study Groups

Of 97 patients assessed for eligibility, 89 patients were included in the study, 4 were excluded due to not meeting inclusion criteria, and 4 declined to participate. Of the 89 patients included, 45 were randomized to the PAR group and 44 were randomized to the ALG group.

**Figure 1:**
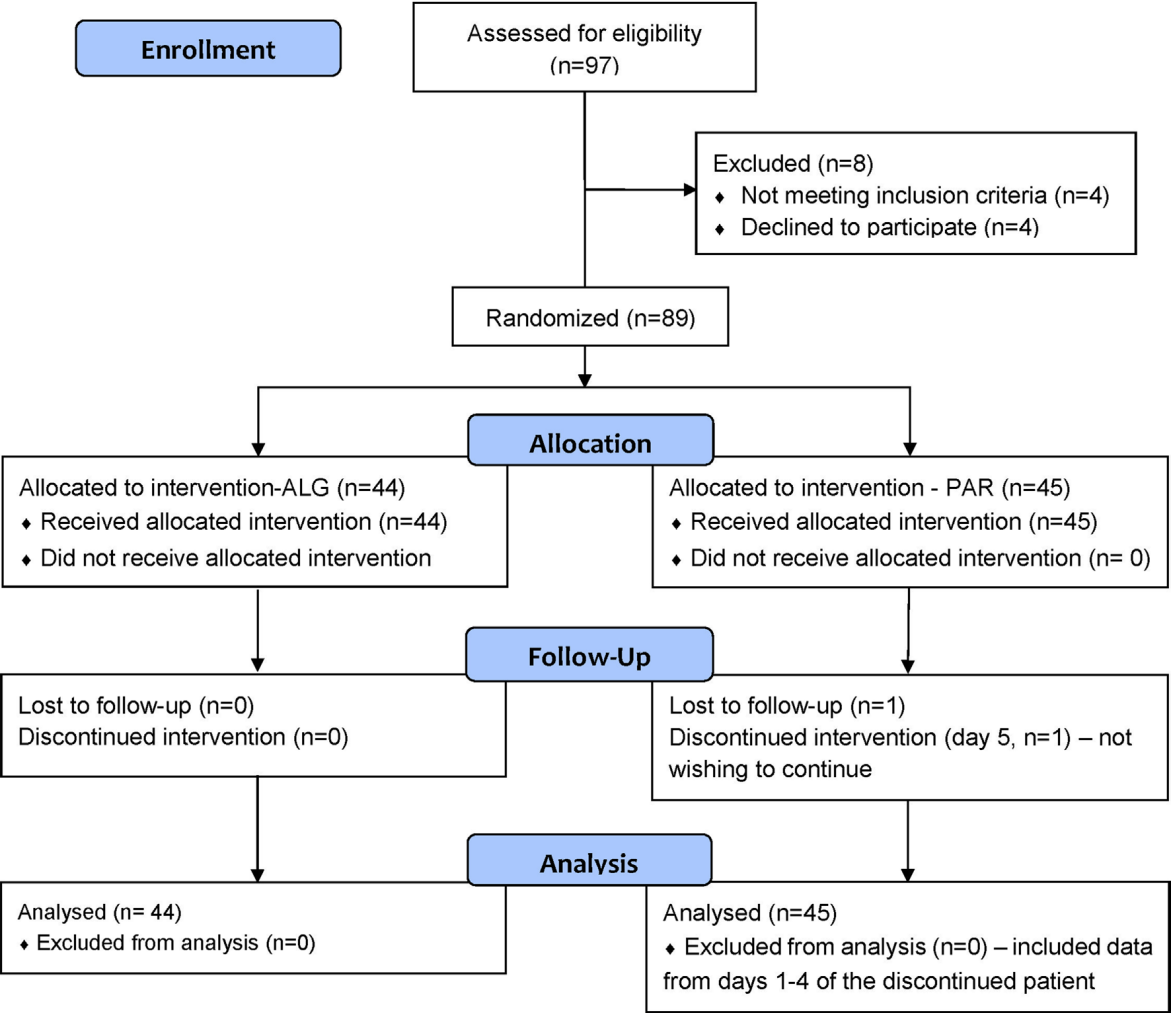
Flow diagram of the progress of the patients through the phases of the trial, according to CONSORT 2010.

The flow diagram of the progress of the patients through the phases of the trial is presented in **Figure 1**. One patient of the PAR group discontinued the study after the first 4 days of medication, without providing any reason.

Patient characteristics were comparable between the study groups (**Table 1**). No significant differences were found between the main demographic characteristics: age (*t* test, *p* = 0.88), gender distribution (chi-square test, *p* = 0.12), weight (*t* test, *p* = 0.46), as well as clinically relevant aspects such as the mean baseline pain intensity, expressed as VAS(PI) score (*t* test, *p* = 0.37).

**Table 1:** Demographics and baseline Visual Analog Pain Intensity [VAS(PI)] scores of the two treatment groups.

Parameter	Treatment		*p* value
	PAR	ALG	
Subjects (*n*)	45	44	−
Male/female (*n*/*n*)	16/29	21/23	0.12 (ns)*
Age (years)	47.38 ± 12.51	46.94 ± 11.42	0.88 (ns)
Weight (kg)	76.88 ± 25.13	80.51 ± 19.23	0.46 (ns)
Height (cm)	163.14 ± 22.22	166.55 ± 18.07	0.45 (ns)
Baseline VAS(PI) score	62.26 ± 13.83	59.31 ± 12.77	0.37 (ns)

*ns, nonsignificant; PAR, acetaminophen; ALG, Algopirin^®^.

For the numerical parameters, mean value ± SD is presented. Comparisons were performed using chi-square test for gender distribution and Student’s t test for the other parameters (significance level, p < 0.05).

In order to estimate a possible effect of age on the conclusion concerning equivalence of the two treatments, comparison of mean pain curves was performed separately for group of subjects under 50 years (52.5%) and group of subjects with age greater than 50 years (47.5%). The results were not significantly different from those for the whole group.

The gender analysis of the efficacy results using *t* test led to the following results: Mean Pain Score Female vs. Male ALG (*p* = 0.49), Mean Pain Score Female ALG vs. PAR (*p* = 0.25), Mean Pain Score Male ALG vs. PAR (*p* = 0.74), and Mean Pain Score Female vs. Male PAR (*p* = 0.75); therefore, it was concluded that gender does not significantly influence the efficacy results.

### Efficacy Results

Effects of the two compared products were analyzed as functions of four variables:

P=P(h, d, i, tr)

where *h* = 0, 1, 2, 4, 6 represent the time in hours after the current administration; *d* = 1, 2, 3, 4, 5 represent the day since the beginning of the treatment; *i* = 1,…, *n_tr_* represents the subjects within a treatment group; *tr* depicts the treatment (PAR and ALG, respectively).

#### Single-Dose Effect *P* = *P*(*h*,*1*,*i*,*tr*)

**Figure 2:**
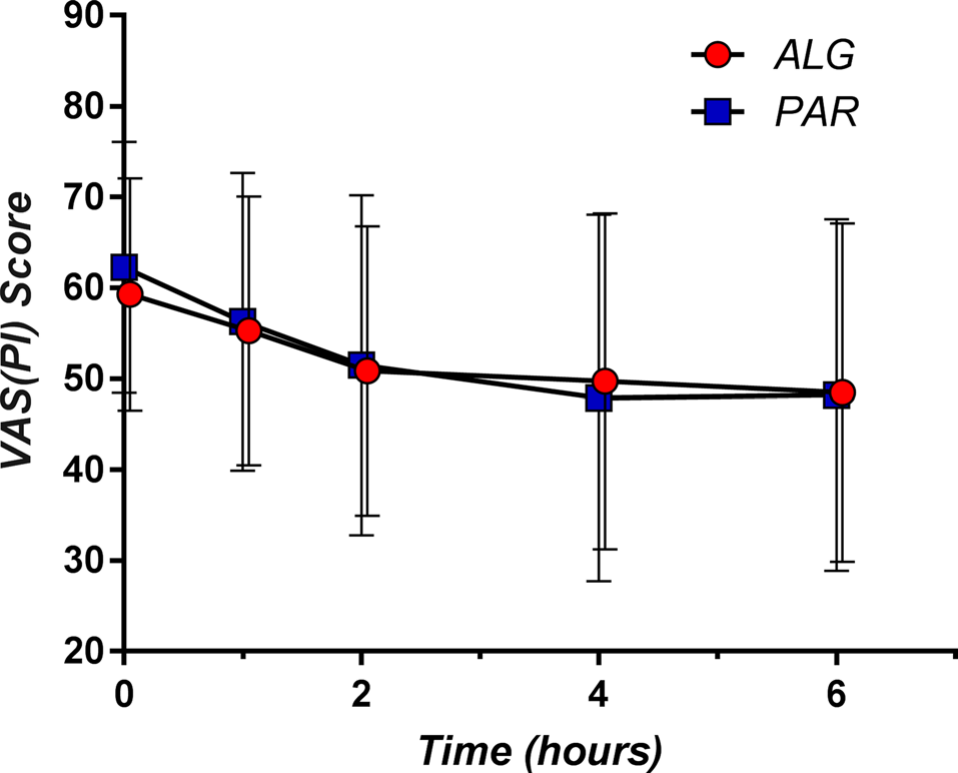
The pain curves (mean ± SD) obtained after single dose administration of Algopirin^®^ (ALG) and acetaminophen (PAR).

The single-dose effect of the trial medication was evaluated from the data obtained after the first administration in the first day of the study. Pain relief relative to baseline was observed in both study groups. It is noteworthy that both ALG and PAR followed similar pain relief patterns: a more pronounced effect within the first 2 h after drug intake continued with a much slower decline (almost a plateau region) of the pain score for up to 6 h (**Figure 2**).

**Figure 3:**
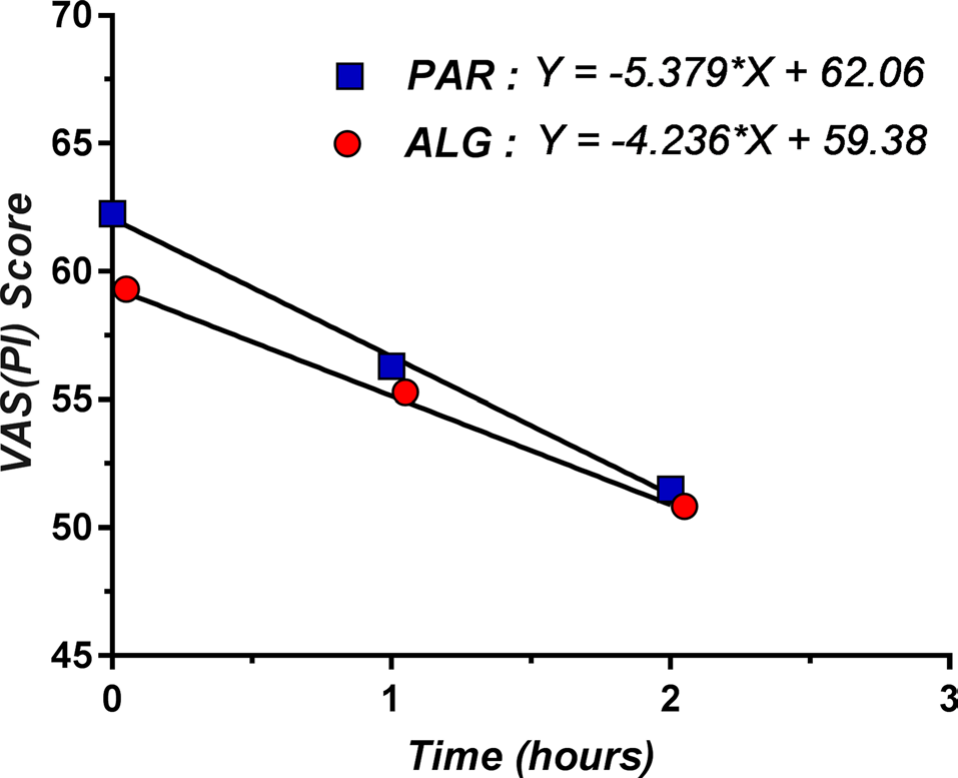
Linear decrease model for pain relief within the first 2 h after single dose administration of ALG and PAR.

Within the first 2 h, the pain relief followed a linear decrease model for both investigated products (**Figure 3**).

Comparison between the mean VAS(PI) score values for the two treatments before and at 1, 2, 4, and 6 h after drug intake was performed using Student’s *t* test (**Table 2**).

**Table 2:** Comparative evaluation of the mean pain score values after single dose administration of the two evaluated treatments, using Student’s *t* test.

Time (h)	ALG group		PAR group		*p* value
	Mean	SD	Mean	SD	
0	59.31	12.77	62.26	13.83	0.37
1	55.28	14.78	56.29	16.38	0.79
2	50.83	15.92	51.50	18.72	0.88
4	49.72	18.48	47.90	20.16	0.70
6	48.47	18.62	48.23	19.35	0.96

Significance level, p < 0.05.

A small (but not significant, *p* = 0.37) difference of the baseline pain score disappeared within 1 h after the administration of the study medication. From this point onwards, the pain score values became practically equal (*p* > 0.70 in all cases). It should also be noted that the SD values were very consistent between the two treatments, suggesting no differences in variability.

#### Multiple-Dose Effects

Regardless of the used treatment, LBP is not a pain to “disappear” within a very short time frame. The mean pain relief at 6 h after the first administration was approximately 20% of the baseline pain (18.27% for ALG and 22.54% for PAR). Therefore, multiple-dose treatment is being required to achieve a clinically meaningful effect.

**Figure 4:**
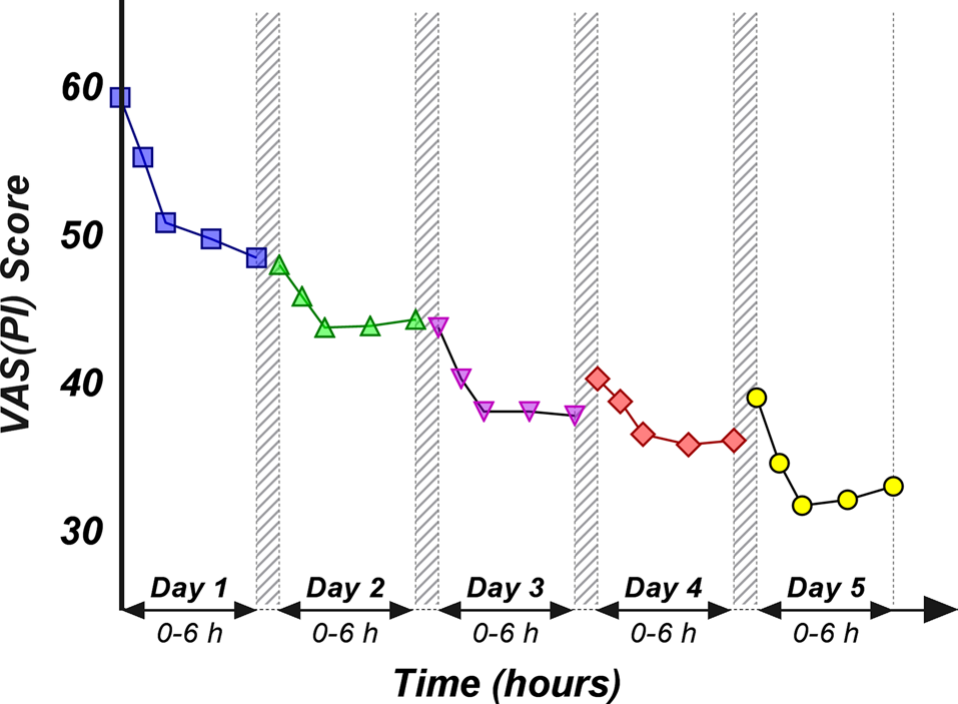
Daily evolution of the mean pain score values within the first 6 h after the morning dose drug administration, evaluated for the first 5 days.

An obvious decrease in the pain score values was obtained as treatment progressed (**Figure 4**). However, it is to note that the slope of the decrease within the first 2 h after morning dose intake appears to be smaller by the day. A pattern can be observed from the graphical representation of the ALG daily pain curves: following the initial pain relief, a subsequent increase of the pain score occurs, but up to a lower value than the previous day’s baseline (**Figure 4**).

**Figure 5:**
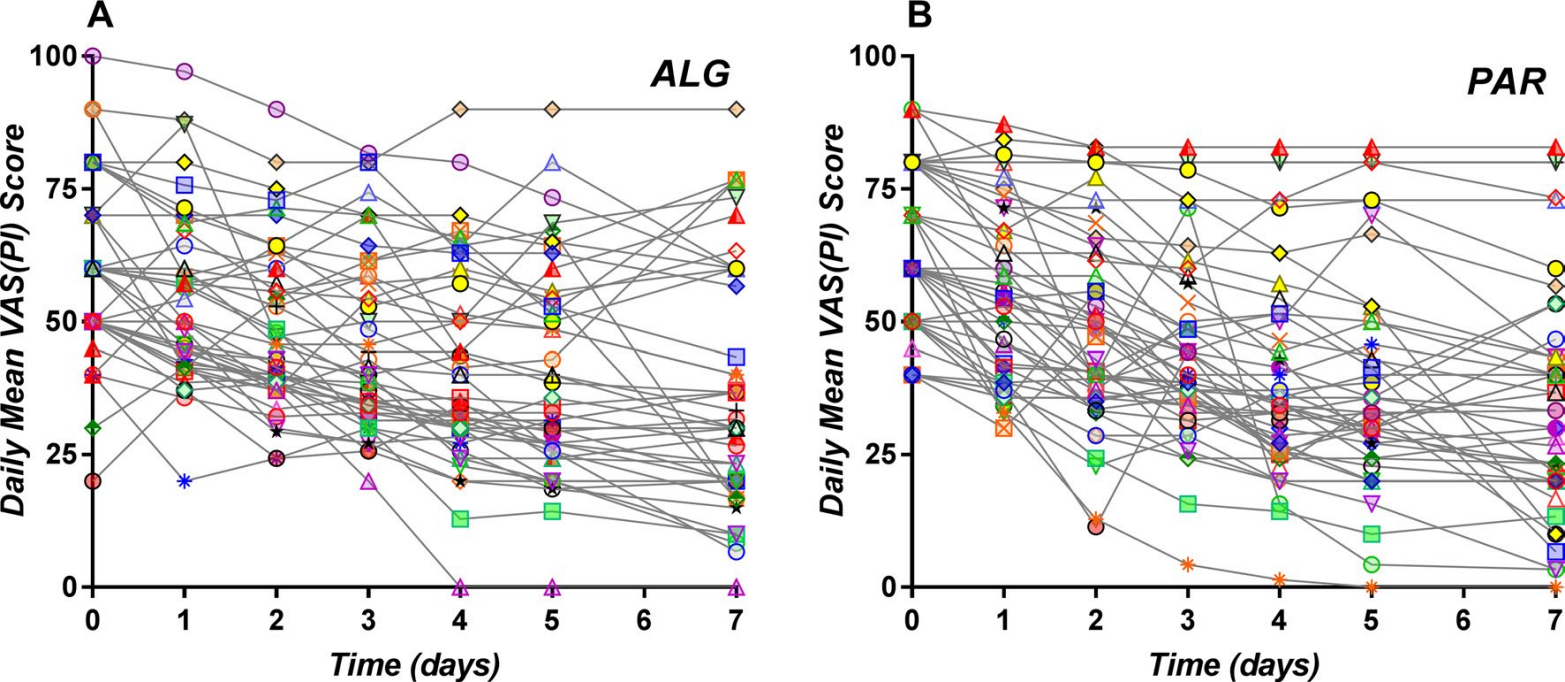
Daily individual pain curves obtained following averaging of all pain score values registered within the same day for the ALG **(A)** and PAR **(B)** treatments.

Daily individual curves obtained following averaging of all pain score values registered within the same day $\bar{P}(.,\;d,i,PAR)$ and $\bar{P}(.,d\;,i,\;ALG)$ are depicted in **Figure 5**. They appear to be homogenously distributed, with no tendency to separate in different clusters.

For further analysis, we evaluated the daily mean pain score for each treatment, by averaging all the pain score values obtained for all patients receiving the specified treatment within each day, $\bar{\bar{P}}(.,\;d,.,tr)$:

$$\begin{array}{l} \bar{P_{.d\;.PAR}}=\sum_{i}\left(\sum_{h}P(h,\;d,i,PAR)/n_{h}\right)/n_{p}\;\\ and\;\bar{\bar{P_{.d\;.ALG}}}=\sum_{i}\left(\sum_{h}P(d,\;h,\;i,\;ALG)/n_{h}\right)/n_{A} \end{array}$$

where *n_A_* and *n_P_* are, respectively, the number of patients who received ALG and PAR.

**Figure 6:**
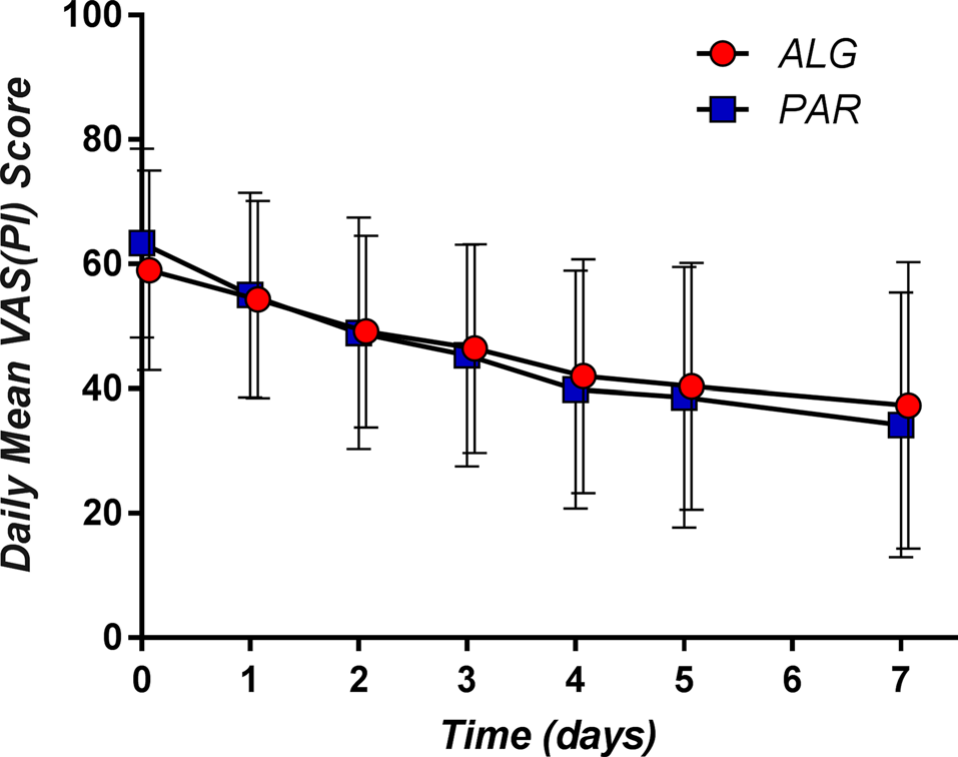
Daily pain curves (mean ± SD) obtained for the two investigated products.

The corresponding mean daily pain curves are presented in **Figure 6**.

The respective differences between the two treatments appeared not to be statistically significant (*p* values between 0.64 in the 4th day and 0.92 on the 2nd day) (**Table 3**).

**Table 3:** Comparative evaluation of the mean daily pain score values of the two evaluated treatments, after three tablets a day administration over five consecutive days.

Day	ALG group		PAR group		*p* value	Mean diff.	95% CI of diff.
	Mean	SD	Mean	SD			
0	59.31	12.77	62.26	13.83	0.37	−2.95	−9.45 to 3.54
1	54.29	15.85	55.72	16.52	0.68	−1.43	−8.21 to 5.35
2	49.12	15.38	49.48	18.80	0.92	−0.35	−7.55 to 6.84
3	46.43	16.74	45.70	18.11	0.84	0.74	−6.57 to 8.04
4	42.01	18.80	40.16	19.48	0.65	1.85	−6.17 to 9.86
5	40.37	19.79	39.19	21.22	0.79	1.18	−7.41 to 9.78

Significance level, p < 0.05.

**Figure 7:**
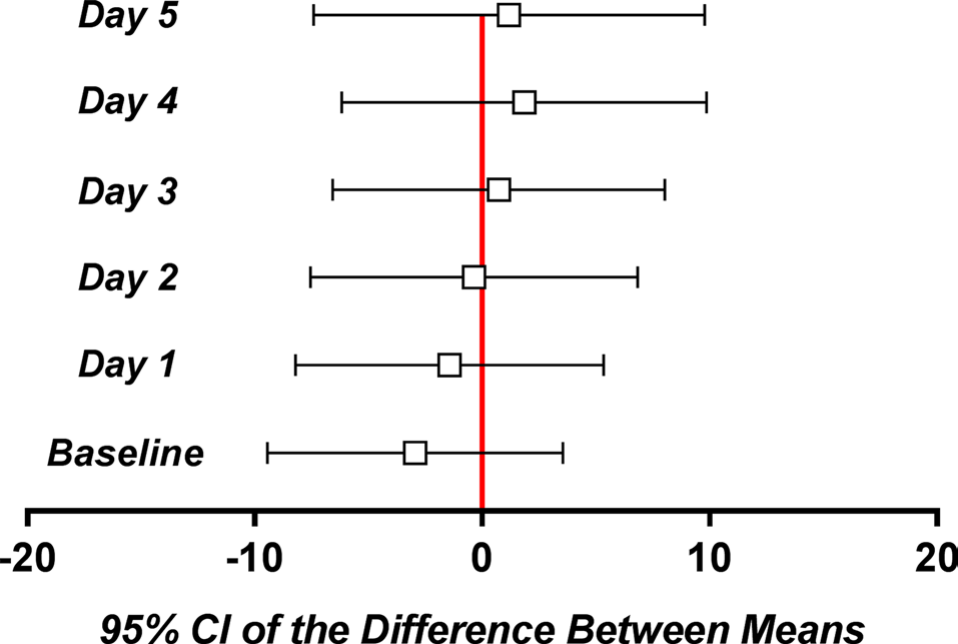
Mean and 95% confidence intervals of the difference between means for the daily pain parameter.

Irrespective of the day, the difference between the mean pain scores was not clinically significant (**Figure 7**).

A common alternative endpoint in the clinical efficacy evaluation of analgesic drugs is the time-weighted Sum of Pain Intensity Differences (SPID), where the pain intensity differences are calculated as the differences between the current pain level and pain at baseline, multiplied by the interval between measurements.

When the length of measuring time intervals tends to zero, SPID becomes the integral of pain curve and equals area under complementary of pain curve that actually represents the effect curve. When time intervals between measuring points remain constant, the parameter becomes the mean of the pain values.

SPID(d,i,tr)=∑h(P(h,d,i,tr)−P(0,d,i,tr))∆hSPID(d,i,tr)=∑hPD(h,d,i,tr)∆h

$$\begin{array}{l} SPID_{0-6h}=PD(1,d,i,tr)\cdot 1+PD(2,d,i,tr)\cdot 1\\ \;+\;PD(3,d,i,tr)\cdot 1+PD(4,d,i,tr)\cdot 1\\ \;+\;PD(6,d,i,tr) \end{array}$$

**Figure 8:**
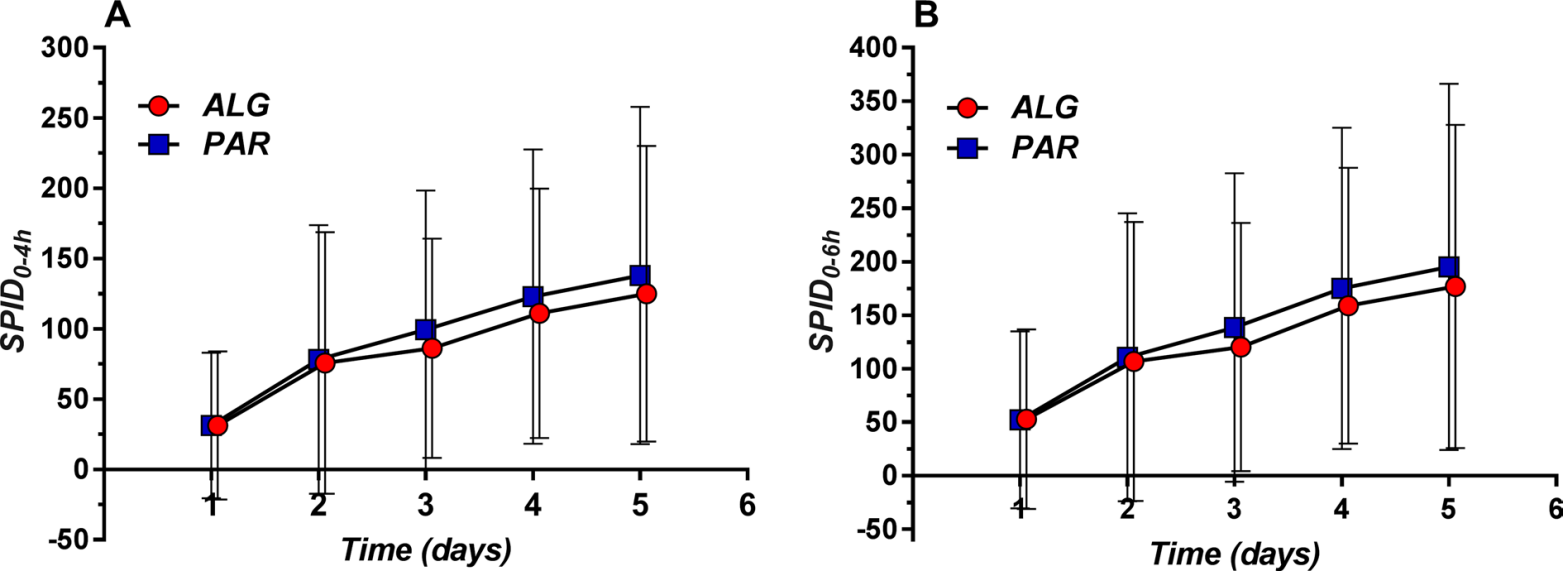
Daily evolution of Sum of Pain Intensity Differences (SPID) (mean ± SD) evaluated within the 0–4 h **(A)** and 0–6 h **(B)** time frame.

Daily means for the 0–4 h interval (SPID_0–4h_) as well for the 0–6 h interval (SPID_0–6h_) were evaluated (**Figure 8**).

The overall mean value was higher for PAR, but the difference analysis found no statistically significant differences (**Table 4**).

**Table 4:** Comparative evaluation of the mean daily Sum of Pain Intensity Differences (SPID) values for the two evaluated treatments, after three tablets a day administration over five consecutive days.

Day	SPID_0–4h_					SPID_0–6h_				
	ALG		PAR		*p* value	ALG		PAR		*p* value
	Mean	SD	Mean	SD		Mean	SD	Mean	SD	
1	31.20	52.74	31.28	51.85	1.00	52.77	83.88	52.33	82.69	0.99
2	75.84	93.18	78.26	95.57	0.90	106.69	130.45	110.58	134.78	0.89
3	86.27	77.91	99.36	99.14	0.50	120.36	116.05	138.66	144.26	0.52
4	111.08	88.79	122.85	104.71	0.55	158.80	129.06	175.17	150.19	0.57
5	124.94	105.26	138.08	120.09	0.50	176.87	151.03	195.17	171.04	0.52
**Mean**	85.87	91.00	93.97	103.54	0.37	123.10	130.89	134.38	147.93	0.38

Significance level, p < 0.05.

In fact, the 5-day difference is less than 10% of the SPID of PAR, and therefore not clinically significant.

Another common endpoint used for the efficacy evaluation of pain drugs is the time to onset of meaningful pain relief, with a value of 50% pain relief being the most utilized threshold (*T*_50_).

**Figure 9:**
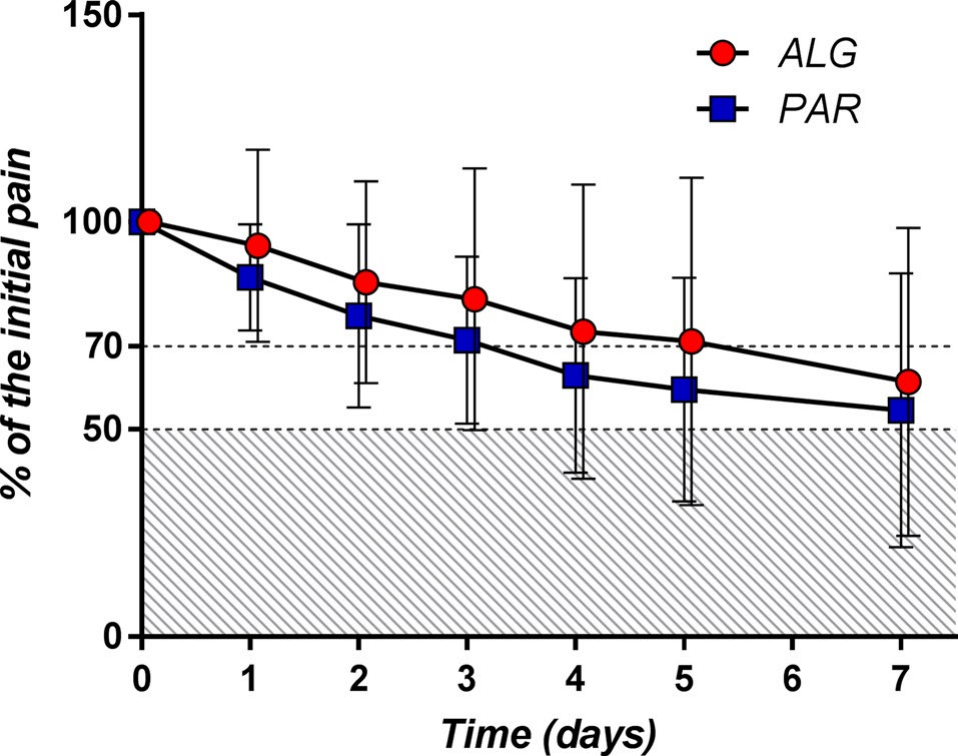
Daily pain curves (mean ± SD) obtained for the two investigated products, expressed as percentage of the baseline pain score values.

*T*_50_ was defined as the time (expressed in days) that contained the first moment when the pain passed the 50% threshold. By examining the daily mean pain scores expressed as percentage of the initial baseline (**Figure 9**), it appears that the mean pain reduction of 50% is an ambitious task that is difficult to achieve (after approximately 3.5 days for PAR and 4.3 days for ALG) (*p* = 0.13).

A pain relief of at least 30% from the baseline value (*T*_70_) objective is more realistic and was achieved after approximately 2 days of treatment for both products (*p* = 0.48).

**Figure 10:**
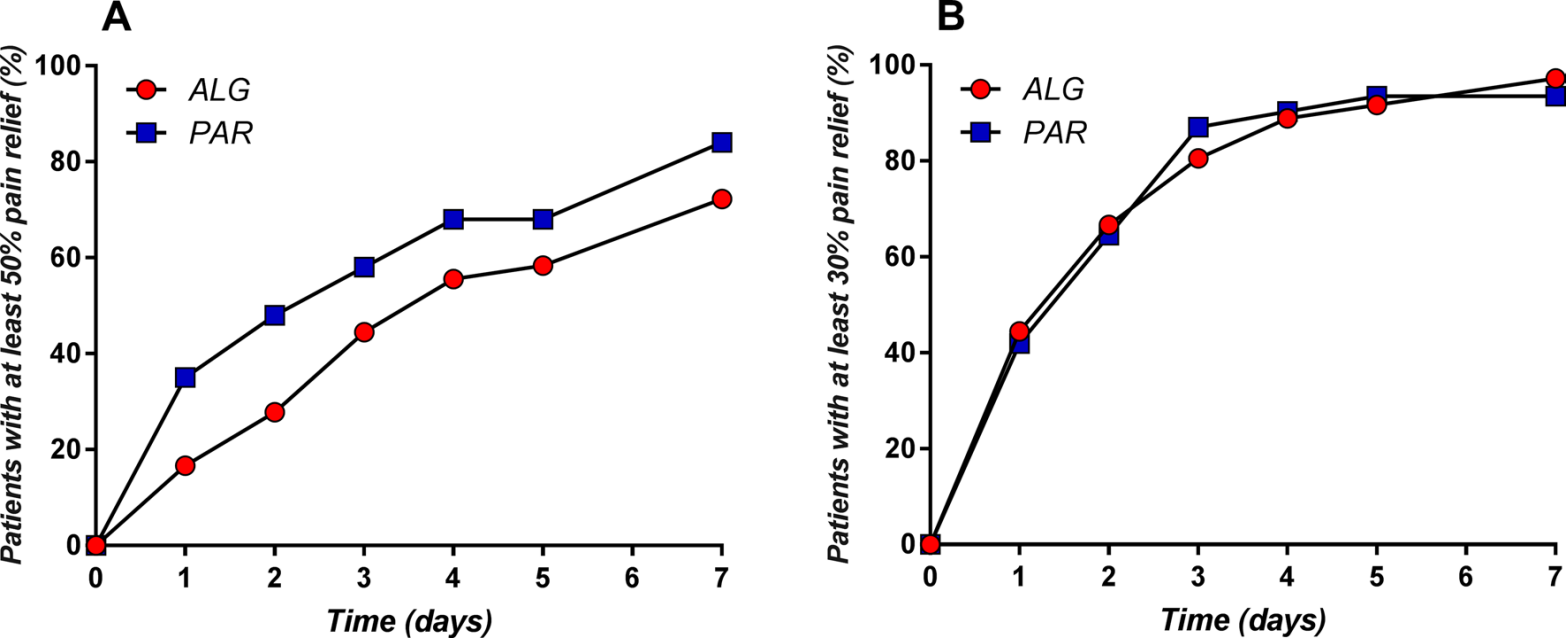
Daily evolution of the percentage of patients with at least 50% **(A)** and at least 30% **(B)** pain relief throughout the treatment.

The evolution of the percentage of patients with at least 50% and at least 30% pain relief throughout the treatment is depicted in **Figure 10**.

The obtained graphs can be considered as “death of pain” curves and their complementary representation relative to the baseline can be viewed as “pain survival curves” and analyzed by means of the survival analysis tools and methods. Similarly, *T*_50_ and *T*_70_ can be considered as “survival time of the pain” values.

Kaplan–Meier estimation of the survival probability at time *t* starting from the ratio between the number of “death cases” *d_i_* and the number of persons observed at time *t_i_* was calculated according to the formula:

$$\hat{S}(t)=\prod_{t_{1}<t}\frac{n_{i}-d_{i}}{n_{i}}$$

In our case, *d_i_* was considered as the additional number of patients with “dead” pain (i.e., with at least 50% pain relief or at least 30% pain relief, respectively).

**Figure 11:**
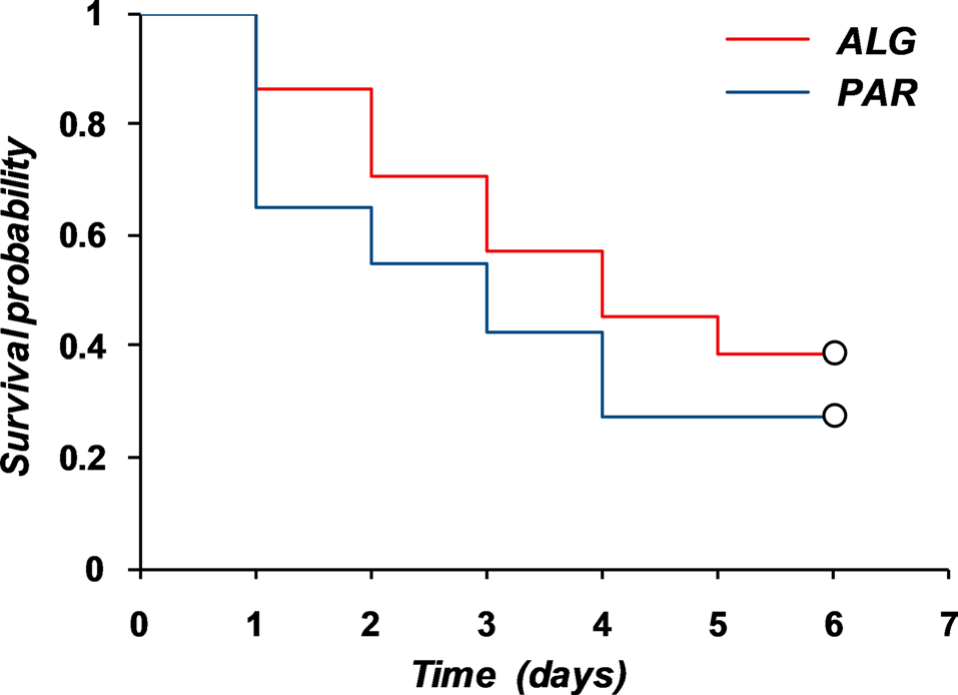
Kaplan–Meier estimations of the pain survival curves.

Graphical representation of the two “pain survival curves” is depicted in **Figure 11**.

Evaluation of the mean survival time of the pain for the two treatments led to the results presented in **Table 5**.

**Table 5:** Comparative evaluation of the time to onset of meaningful pain relief.

Parameter	ALG group		PAR group		*p* value	Mean diff.	95% CI of diff.
	Mean	SD	Mean	SD			
*T*_50_	4.34	2.57	3.48	2.62	0.13	0.85	−0.25 to 1.95
*T*_70_	1.98	1.81	1.74	1.23	0.48	0.23	−0.42 to 0.89

The mean survival time was estimated as the area under the survival curve. Non-parametric statistical tests to compare the two survival curves indicated that the difference between them is not significant (**Table 6**).

**Table 6:** Tests for equality of the survival distribution functions.

Test	Observed value	Critical value	*p* value
Log-rank	2.23	3.84	0.14
Wilcoxon	3.46	3.84	0.06
Tarone–Ware	2.90	3.84	0.09

Degrees of freedom = 1, alpha = 0.05.

Thus, both the parametric and nonparametric comparison of pain curves indicate the equality of the analgesic effect of the two treatments.

## Discussions

### Selection of the Endpoint and Comparator

A series of recent studies (Chiarotto et al., 2015; Chiarotto et al., 2016) provided suggestions on which outcome domains and measurement instruments to use in patients with low back pain (LBP). Six domains were identified as highly relevant: 1) physical functioning, 2) pain intensity, 3) health-related quality of life, 4) work, 5) psychological functioning, and 6) pain interference.

Over time, 75 parameters were discussed. But it is worth mentioning what Professor Douglas Altman reported at the 2016 meeting of the Core Outcome Measures in Efficiency Trials (COMET) “Trials should build on what’s already done; it’s not the place to be too novel; use a core outcome set if it exists; too much originality won’t help patients.”

Hence, it was considered the usual endpoint in clinical trials concerning analgesic drugs: the pain reported by the patient. It is to note that, for the registration of new drug combinations, the current guidelines recommend efficacy evaluations to be performed against all the active components. However, in the particular case of the present drug combination, this approach would lead to a five-study-group clinical trial, which would clearly lack any clinical and ethical justification.

On the other hand, as a result of the continuous efforts for increasing the certainty level of results, the regulatory biostatisticians consider as a gold standard testing the effect of a new drug by comparison against placebo (Temple, 1996; Temple, 1997; Temple and Ellenburg, 2000), which is rather hostile to studies with an active comparator.

As opposed to them, biostatisticians serving in Ethics Committees consider the use of placebo to be unethical, due to unacceptable risks for the patients receiving placebo treatments (Rothmans and Michels, 1994). Furthermore, many consider the arguments about superiority of placebo control studies to be “a collection of myths” (Freedman, 1987, Freedman et al., 1996a; Freedman et al., 1996b).

Numerous guideline documents for the management of LBP in primary care have been published in various countries around the world (Koes et al., 2001; Airaksinen et al., 2006; Koes et al., 2006; van Tulder et al., 2006). They all consider PAR the first-line treatment option, with NSAIDs as alternates.

Despite a wide consensus regarding the clinical use of PAR, two recent clinical trials concluded that its effect was not superior to placebo. However, for these studies, some very “strange” aspects in terms of dose choice, comparator, duration, and goals have to be underlined. One study (Williams et al., 2014) concluded that median time to recovery from LBP was 16 days in the PAR group and 17 days in the placebo group. However, the primary endpoint “time until recovery from low back pain,” with recovery defined as “a pain score of 0 or 1 (on a 0–10 pain scale) sustained for 7 consecutive days” is virtually impossible to obtain, regardless of the treatment.

The other study (Wetzel et al., 2014) concluded that the placebo effect is superior to PAR. The drug was intravenously administered to patients with LBP chronically treated with opiates, a very special category of patients. In fact, both the above studies were subsequently withdrawn after clear conflict of interest of the authors emerged. Interestingly, only these two studies were considered as having “high quality” in a Cochrane report on the use of PAR in LBP (Saragiotto and Machado, 2016), with another 21 clinical trials being considered as “low quality”. It becomes therefore obvious that, at times, statistical significance and clinical significance can drift far away one from the other.

Taking into account all the above considerations, for the assessment of the clinical significance of the analgesic effect for the ASA–PAR–CAF–CLF combination, we considered the best comparator to be the standard analgesic clinical treatment for LBP, i.e., PAR. In fact, in one of our previous experiments (Voicu et al., 2016), the effect of ALG was found to be significantly superior to both ASA and CLF, as evaluated on a carrageenan-induced rat paw model of inflammation, and their further use as comparators in the present study is not being considered justified.

### Safety–Efficacy Balance: Theoretical Considerations

The first question to be answered by this study was whether ALG has a significant clinical effect in LBP. It is obvious that the previously proven effect of an ALG single dose in migraine (similar to Excedrin^®^) cannot be extrapolated to LBP.

**Figure 12:**
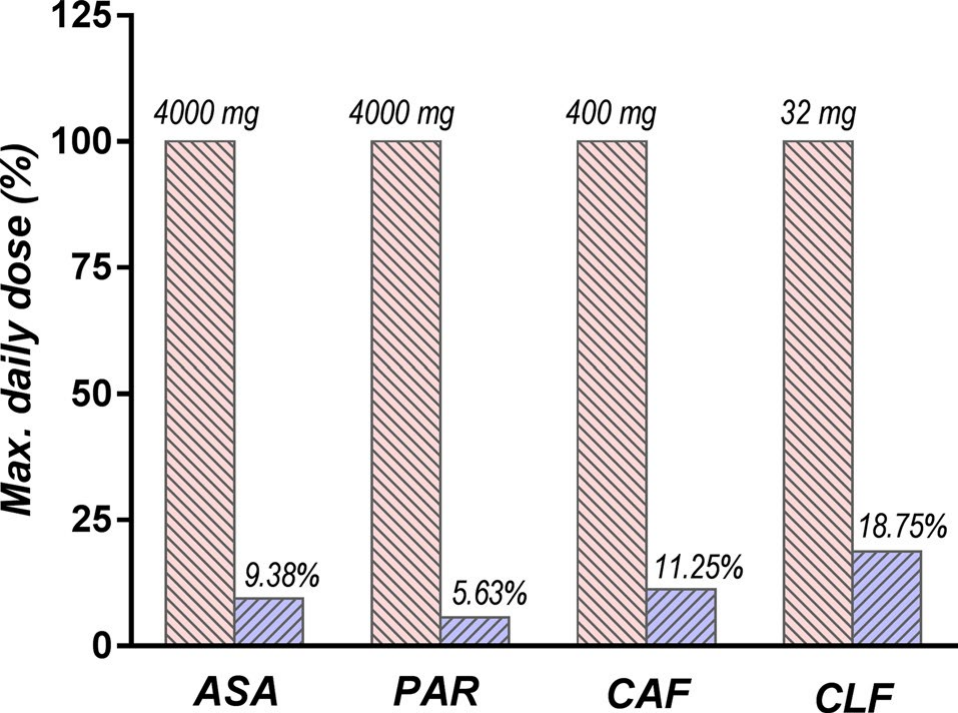
Comparison between daily doses of active components used in the study (depicted in blue) and the maximum doses recommended by the current guidelines (depicted in red).

The main risk of non-efficacy for the investigated product was due to the very low dose of active components in the tested combination, as compared to their usual doses. The amounts corresponding to the daily dose of ALG administered in our experiment was very low (375 mg for ASA, 225 mg for PAR, 45 mg for CAF, and 6 mg for CLF, corresponding to three ALG tablets), ranging between 5.3% and 17.9% of the maximum daily dose of the active components (4,000 mg for ASA and PAR, 400 mg for CAF, and 32 mg for CLF) (Chlorpheniramine Dosage Guide with Precautions, 2019; Summary Acetaminophen Dosage Guide with Precautions, 2019; Summary Aspirin Dosage Guide with Precautions, 2019) (**Figure 12**).

As a result, ALG is expected to be overall much safer than other drugs used in the treatment of LBP and is therefore a viable therapeutic alternative, mainly for patients with gastrointestinal sensibility.

### Pharmacokinetic-Pharmacodynamic (PK-PD) Modeling

The effect of an analgesic formulation expressed as pain score at the *t_k_* moment is not an instantaneous effect, but rather a cumulative one. We defined the effect at time *t_k_* by what we called the “derivative of pain curve” using the formula:

$$\frac{dP}{dt}(t_{k})\approx \frac{n_{k}-n_{k-1}}{t_{k}-t_{k-1}}$$

where *n_k_*_−1_ and *n_k_* are the VAS(PI) scores at moments *t_k_*_−1_ and *t_k_*, respectively.

**Figure 13:**
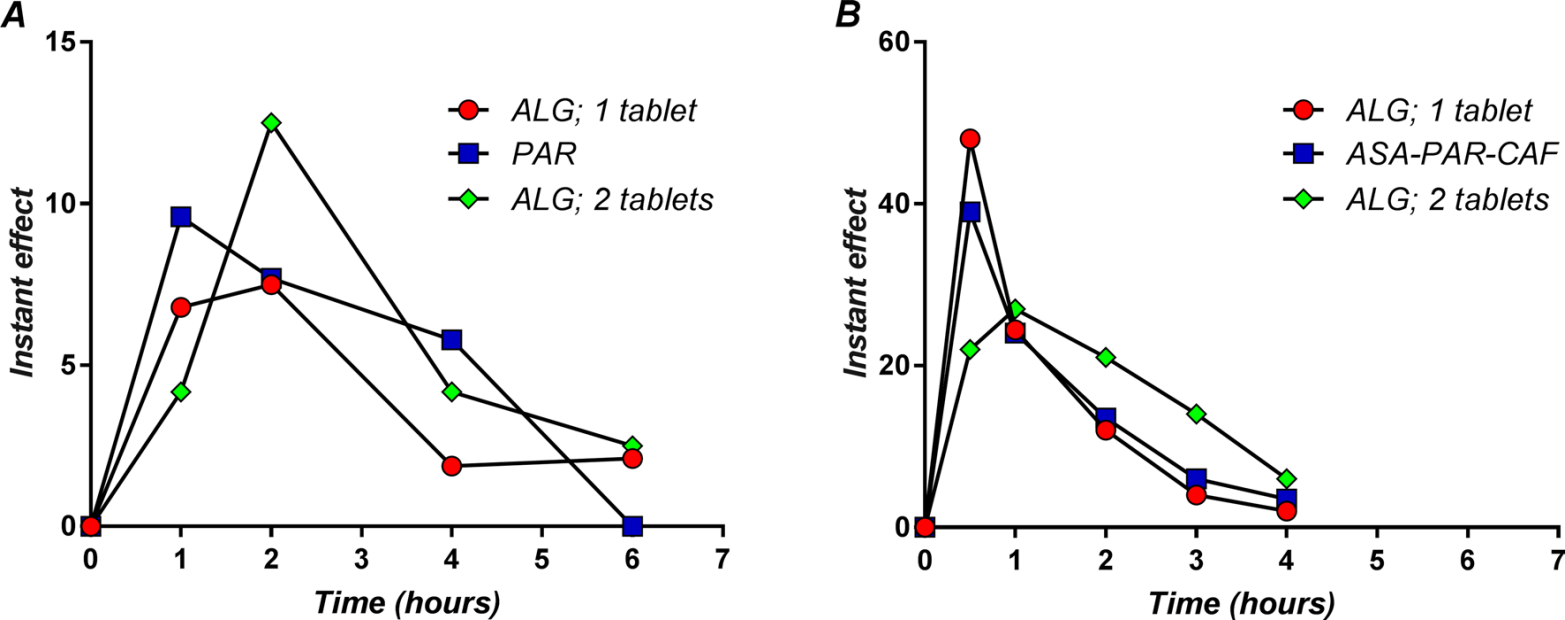
The instant effect after a single dose administration of the study medication in back pain **(A)** and migraine **(B)**.

The derivatives of the mean pain curves after the first dose of medication are presented in **Figure 13A**.

The time course of effect is naturally related to the release kinetics of the active ingredients from the tablets and, finally, to their pharmacokinetics. Pharmacokinetics were considered by analogy with available data on similar combinations in the literature. It is worth noting that, despite ASA and PAR being in therapy for a very long time, and their effect being both well known and well established, there are very few data with reference to their pharmacokinetic profile.

The maximum analgesic effect of ALG in LBP occurred between 1 and 2 h (**Figure 13A**). It is to note that the maximum analgesic effect in LBP appears to be delayed with about half an hour and approximately 10 times lower in intensity than the ALG effect in migraine (Voicu et al., 2017b) (**Figure 13B**). The peak plasma concentrations of ASA and PAR, the main active components of ALG, were reported as 15 min and 30 min (Muir et al., 1997). As a function of formulations, these values may be somewhat higher, but not more than double (Kanani et al., 2015). Thus, there is a delay between peak plasma concentrations of active components and the maximum analgesic effect.

The PAR effect occurred a little earlier than the ALG effect, but the ALG effect has been shown to be influenced by the dose. In a previous pilot clinical trial (data not published), two ALG tablets were administered to five subjects, and the maximum effect occurred at the first measurement point, demonstrating a faster coupling between plasma levels and effect. The maximum effect of two ALG tablets appeared visually greater than that of PAR, but the low number of patients administered with two ALG tablets could not allow a significant statistical analysis.

### Outliers

**Figure 14:**
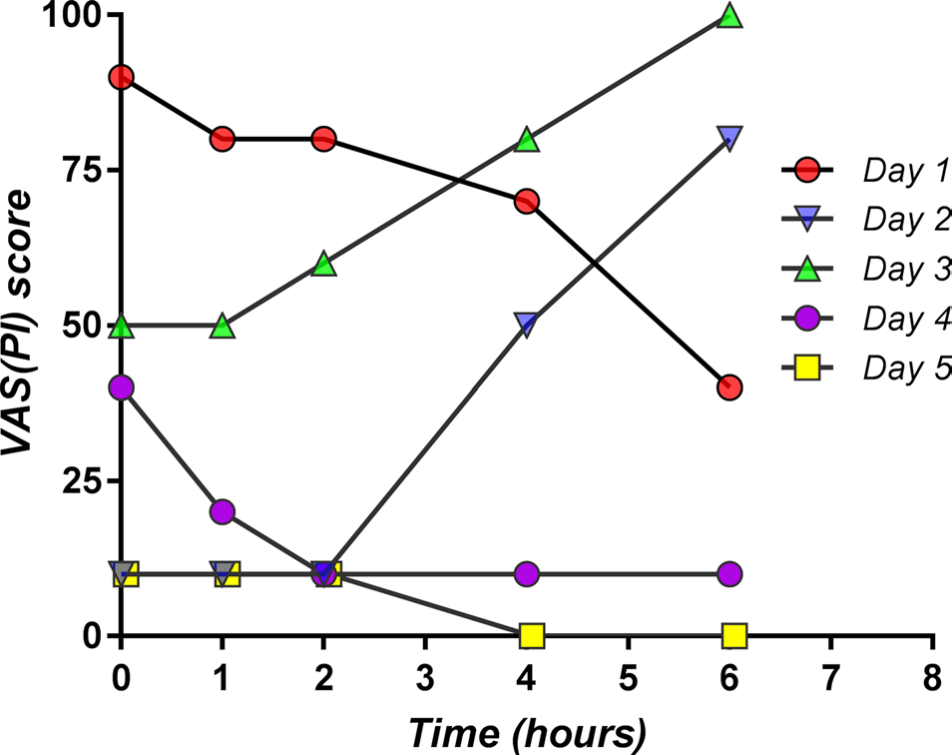
Individual pain curves for five consecutive days obtained from one patient participating in the study, which exemplifies the reversal of drug effect related to physical effort (day 2 and day 3).

An important risk of failure during the study was the relationship between pain and effort. After a significant decrease of the baseline pain after the study medication was administered, the patient might feel “healed” and be tempted to resume the physical effort that the pain has prevented him from having. Thus, following an initial pain relief, a sudden increase may occur, which, in fact, cannot be considered as a result of the treatment. An actual example obtained from one of the study patients is depicted in **Figure 14**.

After the “expected” analgesic effect on the first day, on the second day, and on the third day of the study, due to the patient undertaking physical effort, a significant increase of the pain level occurred, unrelated to medication. In the next 2 days, the effect of the medication appeared again to be “expected,” and the pain was practically gone. The patient is therefore an “outlier.”

The question of whether outlier values should be kept or dropped is very difficult, for sometimes their impact on the results of the analysis could be determinant for the conclusions of the study (Mircioiu et al., 2010). Fortunately, in the present study, the conclusion regarding the equivalence of the analgesic effects of the two treatments was the same in both types of analysis (with and without outliers).

### Choice of the Statistical Methods

A final discussion on the statistical methods to compare the effects of the two treatments should be made. There is still a never-ending debate among statisticians whether parametric or of nonparametric methods have greater suitability for data analysis. A recent paper on this subject (Mircioiu and Atkinson, 2017) concluded that, at least in the case of biological data, a reasonable approach would be to apply both types of tests and to compare the results. If there is a good correlation between the conclusions, the result can be considered to be sufficiently reliable (Purcaru et al., 2010; Mircioiu et al., 2013). In the case of non-correlation, a more detailed analysis of the data structure is needed (Preda et al., 2012).

Comparison of the pain scores at different measurement points as well as pain curve parameters, such as SPID values, were performed on the assumption of a normal distribution of these parameters. On the other hand, because the distribution of proportions of patients with at least 50% pain relief is not known, and there are no arguments to assume a normal distribution, their comparison was performed using the log-rank test. Other authors used the same log-rank test in the statistical analysis of analgesic formulations (Lipton et al., 1998; Hu et al., 1999; Wenzel et al., 2003; Goldstein et al., 2006; Reddy, 2013). Regardless of the tests applied, the effects of the treatment appeared to be equivalent; thus, this conclusion is highly reliable.

### Evolution of Disability Measured by the Roland–Morris Questionnaire

The Roland–Morris Questionnaire (RMQ) is a self-rated physical disability measure on a 24-point scale (Stratford et al., 1996). Testing was considered to have a low sensitivity for comparing treatments but was used to highlight the effects of treatments over time on patient disability.

**Figure 15:**
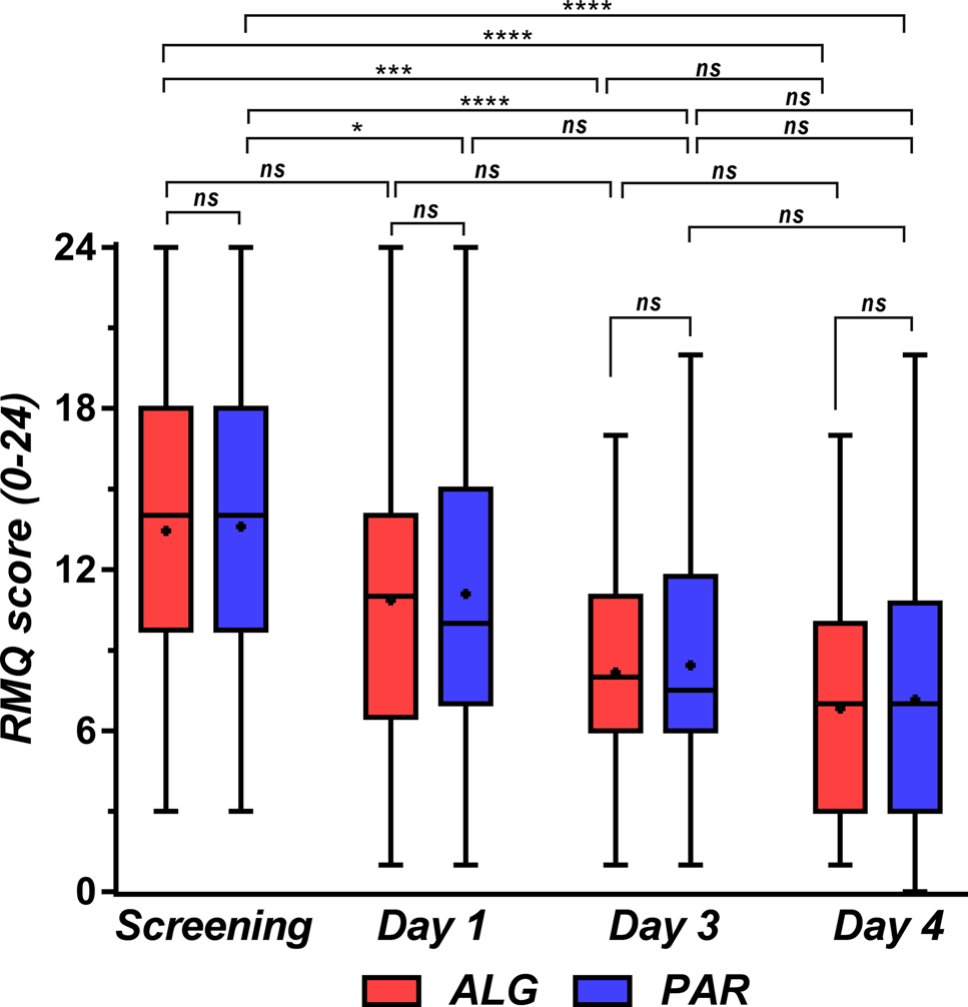
Roland–Morris disability score at the initial screening and in treatment days 1, 3, and 4. Data are presented as median and lower limit, 25th, 75th, and upper limit percentiles. One-way ANOVA with *post hoc* Tukey HSD (honestly significant difference) multiple comparison test was used for comparison of data sets. Levels of significance: ns, nonsignificant (*p* > 0.05), *p ≤ 0.05, **p ≤ 0.01, ***p ≤ 0.001, ****p ≤ 0.0001.

The score did not differ significantly between treatments, but after 4 days, there was a drop of half the initial value. ALG appears to have a greater efficacy, but the difference is not statistically significant (**Figure 15**).

## Conclusions

The time course of the analgesic effect of ALG and PAR was very similar. The effect occurs quickly and increases continuously for 4 h. After this time, analgesia decreases up to 8 h, and the pain level is maintained below the baseline. Over a week of treatment, the mean daily pain decreased continuously, in a linear fashion over the first 3 days and asymptotically in the second interval. The mean pain values per hour after the first dose as well as the mean daily pain values were compared using Student’s *t* test. The conclusion was that the differences between the effects of the two treatments were not statistically significant (*p* > 0.3). The same conclusion was drawn from comparing the daily sums of pain intensity differences SPID_0–4h_ and SPID_0–6h_ Daily proportions of patients with at least 50% pain relief revealed differences between the two treatments, but the differences were not statistically significant. Comparison of pain survival curves using the log-rank test indicated that the hazards rate is not significantly different from the unit (*p* = 0.06).

An alternative treatment that starts with two ALG tablets following the administration schedule of two tablets at the first administration, on the first day, followed by one tablet every 8 h for the rest of the study period leads to significantly better effects (data not shown). Correlation with the pharmacokinetics of ASA has shown a delay of about half an hour between the maximum plasma level and the maximum effect. The pattern of derivatives of pain curves after the first dose administration of ALG and PAR was very similar and much more similar to the ALG differential model of migraine analgesia found in a previous study.

As ASA, PAR, and CAF doses are very low in ALG, this combination should be considered as an alternative treatment for LBP, mainly for patients with gastrointestinal and hepatic sensitivity.

## Ethics Statement

The study protocol (EudraCT number: 2015-002314-74) was approved by the National Agency for Medicines and Medical Devices (approval number 30523E/04.04.2016) and the National Bioethics Committee for Medicines and Medical Devices (approval number 124/2016). All participants gave written informed consent prior to study participation and were instructed by specialized personnel on how to record the characteristics of their back pain. All patients were allowed to terminate their participation in the trial at any time, without restrictions.

## Author Contributions

VV coordinated the entire project; CM elaborated the clinical trial design and performed the statistical analysis; CP, MJ, and VB were clinical investigators; RS and AC performed data collection, built and structured the clinical study results database, and performed statistical analysis; VA was responsible for the quality assurance and IM was responsible for production of the investigational drugs and study monitoring; CM, IM, and VA prepared the manuscript. All authors approved the manuscript.

## Funding

Finalization of this work was supported by the PNCDI II grant 139/2014 of the Romanian Ministry of Education and Research. Publication of this paper was financially supported by “Carol Davila” University of Medicine and Pharmacy through Contract no. 23PFE/17.10.2018 funded by the Ministry of Research and Innovation within PNCDI III, Program 1 – Development of the National RD system, Subprogram 1.2 – Institutional Performance – RDI excellence funding projects.

## Conflict of Interest Statement

AC was employed by CEBIS International, Bucharest. The company had no role in the design of the study, in the collection, analyses, or interpretation of data, in the writing of the manuscript, and in the decision to publish the results.

The remaining authors declare that the research was conducted in the absence of any commercial or financial relationships that could be construed as a potential conflict of interest.
